# Lead Induces Mitochondrial Dysregulation in SH-SY5Y Neuroblastoma Cells via a lncRNA/circRNA–miRNA–mRNA Interdependent Networks

**DOI:** 10.3390/ijms26146851

**Published:** 2025-07-17

**Authors:** Yu Wang, Xuefeng Shen, Ruili Guan, Zaihua Zhao, Tao Wang, Yang Zhou, Xiaoming Chen, Jianbin Zhang, Wenjing Luo, Kejun Du

**Affiliations:** 1 Department of Occupational and Environmental Health and Ministry of Education Key Lab of Hazard Assessment and Control in Special Operational Environment, School of Public Health, Fourth Military Medical University, Xi’an 710032, China; cd8848@fmmu.edu.cn (Y.W.); xfshen@fmmu.edu.cn (X.S.); guanruili@fmmu.edu.cn (R.G.); zaihuazhao@fmmu.edu.cn (Z.Z.); taowang@fmmu.edu.cn (T.W.); zhouyang@fmmu.edu.cn (Y.Z.); xiaomingchen@fmmu.edu.cn (X.C.); zjbin777@fmmu.edu.cn (J.Z.); 2 School of Public Health, Shanxi University of Chinese Medicine, Xianyang 712046, China

**Keywords:** lead, mitochondrial, lncRNA, circRNA, miRNA

## Abstract

Lead (Pb) exposure poses a significant public health concern due to its neurotoxic effects. While mitochondrial dysfunction is implicated in lead neurotoxicity, the precise molecular mechanisms, particularly the role of non-coding RNA-mediated competing endogenous RNA networks, remain underexplored. SH-SY5Y neuroblastoma cells were treated with 10 μM lead acetate. Cell viability was assessed by Cell Counting Kit-8 (CCK-8). Mitochondrial ultrastructure and quantity were analyzed via transmission electron microscopy (TEM). Key mitochondrial dynamics proteins were examined by Western blot. Comprehensive transcriptome sequencing, including long non-coding RNAs (lncRNAs), circular RNAs (circRNAs), microRNAs (miRNAs) and mRNAs, was performed followed by functional enrichment and ceRNA network construction. Selected RNAs and hub genes were validated using quantitative real-time reverse transcription polymerase chain reaction (qRT-PCR). Lead exposure significantly reduced SH-SY5Y cell viability and induced mitochondrial damage (decreased quantity, swelling, fragmentation). Western blot confirmed an imbalance in mitochondrial dynamics, as indicated by decreased mitofusin 2 (MFN2), increased total and phosphorylated dynamin-related protein 1 (DRP1). Transcriptomic analysis revealed widespread differential expression of lncRNAs, circRNAs, miRNAs, and mRNAs. Enrichment analysis highlighted mitochondrial function and oxidative stress pathways. A ceRNA network identified five key hub genes: *SLC7A11*, *FOS*, *HMOX1*, *HGF*, and *NR4A1*. All validated RNA and hub gene expression patterns were consistent with sequencing results. Our study demonstrates that lead exposure significantly impairs mitochondrial quantity and morphology in SH-SY5Y cells, likely via disrupted mitochondrial dynamics. We reveal the potential regulatory mechanisms of lead-induced neurotoxicity involving ceRNA networks, identifying hub genes crucial for cellular stress response. This research provides a foundational framework for developing therapeutic strategies against lead-induced neurotoxicity.

## 1. Background

Lead (Pb) is a pervasive environmental toxin arising from diverse sources such as industrial emissions, fossil fuel combustion, and the widespread use of lead-containing materials, including batteries and paints [1]. With the rapid pace of urbanization, lead exposure is recognized as a significant global public health challenge [2]. Both chronic and acute lead exposure have been strongly linked to cognitive impairment and an increased risk of neurodegenerative diseases, as evidenced by numerous epidemiological studies [3]. Our previous research using a lead-exposed mouse model further corroborated the neurotoxic effects of lead by demonstrating significant deficits in learning and memory. Despite extensive documentation of lead’s detrimental impacts, the precise cellular and molecular mechanisms underlying lead-induced neurotoxicity, particularly those affecting mitochondrial structure and function in neural cells, remain poorly understood.

Mitochondria play a central role in maintaining neuronal health, functioning not only as the primary source of adenosine triphosphate (ATP) but also as regulators of calcium homeostasis, apoptotic signaling, and reactive oxygen species (ROS) production [4]. Mounting evidence indicates that mitochondrial dysfunction is a key pathological feature of various neurodegenerative conditions, including Alzheimer’s and Parkinson’s diseases [5,6,7]. The dynamic equilibrium between mitochondrial fission and fusion, primarily governed by guanosine triphosphatases (GTPases) such as mitofusin 1/2 (MFN1, MFN2) and dynamin-related protein 1 (DRP1), is essential for sustaining mitochondrial and neuronal integrity [8,9]. This balance is tightly controlled by post-translational modifications, such as DRP1 phosphorylation at Ser616 by kinases such as c-Jun N-terminal kinase (JNK), and by autophagy-related proteins such as microtubule-associated protein 1 light chain 3 beta (LC3B), which are involved in mitochondrial quality control [10,11,12]. Disruption of these regulatory pathways compromises mitochondrial homeostasis and can trigger neuronal injury, ultimately contributing to the development of neurodegenerative disorders [12,13,14,15]. Recent studies have reported that lead exposure can disturb mitochondrial dynamics and function in neuronal cells [16], including SH-SY5Y neuroblastoma cells, leading to oxidative stress, reduced cell viability, and ultrastructural damage [17]. However, the potential contribution of non-coding RNAs (ncRNAs), including long non-coding RNAs (lncRNAs), circular RNAs (circRNAs), and microRNAs (miRNAs), to lead-induced mitochondrial dysregulation remains largely unexplored. Therefore, the present study aims to comprehensively elucidate the molecular and transcriptomic alterations in SH-SY5Y cells following lead exposure, focusing on mitochondrial dynamics, key regulatory proteins, and ncRNA-mediated mechanisms, thereby providing new insights into the pathogenesis of lead-induced neurotoxicity.

In recent years, accumulating evidence has revealed that ncRNAs play crucial roles in regulating neuronal function and maintaining mitochondrial homeostasis [18,19]. These ncRNAs are integral to gene expression networks that govern neuronal survival, synaptic plasticity, and cellular stress responses [20,21,22]. Notably, lncRNAs and circRNAs can function as components of the competing endogenous RNA (ceRNA) network, acting as molecular sponges for miRNAs and thereby indirectly regulating the translation of key mitochondrial regulatory genes [23,24]. For instance, mitochondrial-encoded lncRNAs, such as *lncMtDloop*, are essential for preserving mitochondrial integrity, and their dysregulation has been closely linked to mitochondrial dysfunction and neurodegenerative disease progression [25]. Moreover, environmental toxicants, including heavy metals such as lead, have been shown to alter the expression profiles of multiple ncRNAs in the brain, contributing to neurotoxicity and the development of neurodegenerative disorders [26]. Despite these advances, the specific roles and regulatory mechanisms of ncRNAs and ceRNA networks in lead-induced mitochondrial dysfunction in neural cells have not been systematically investigated, especially at the transcriptomic level.

The SH-SY5Y neuroblastoma cell line is widely employed as an in vitro model to investigate neuronal toxicity and mitochondrial dysfunction due to its neuronal characteristics and high sensitivity to environmental toxins [27,28,29]. Previous studies using SH-SY5Y cells have shown that lead exposure results in pronounced mitochondrial dysfunction, characterized by loss of mitochondrial membrane potential, increased oxidative stress, and decreased cell viability [30,31,32]. Nevertheless, most existing research has primarily focused on functional and morphological alterations, with limited exploration of the underlying molecular mechanisms. Specifically, the precise regulatory roles of ncRNAs in controlling mitochondrial fusion and fission machinery, their upstream signaling events, and the involvement of autophagy-related proteins in lead-induced neurotoxicity remain largely uninvestigated.

In this study, we aim to conduct a comprehensive ceRNA network analysis to investigate how lead exposure affects the expression of lncRNAs, miRNAs, and mRNAs in SH-SY5Y cells, with the goal of uncovering underlying molecular mechanisms. We hypothesize that lead exposure disrupts mitochondrial quantity and function by modulating relevant signaling pathways, ultimately impairing neuronal physiological functions.

## 2. Results

### 2.1. Effects of Lead Acetate on SY5Y Cell Viability and the Number of Mitochondrial

**Figure 1:**
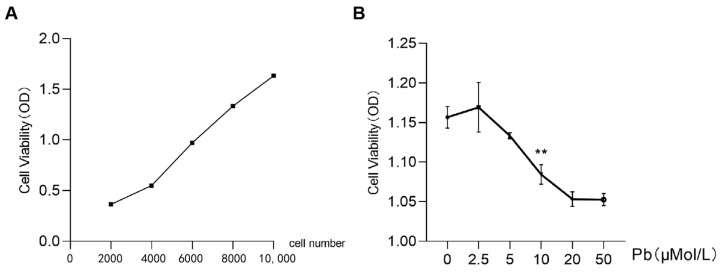
The effects of Pb on SH-SY5Y cells. (**A**) Cell growth curve; (**B**) cell vitality of SH-SY5Y cells treated by different doses of Pb. Results are expressed as mean ± SEM of at least three independent experiments. Control and 10 μmol/L Pb exposure, statistically different, are marked by asterisk (*n* = 3, ** *p* < 0.01).

**Figure 2:**
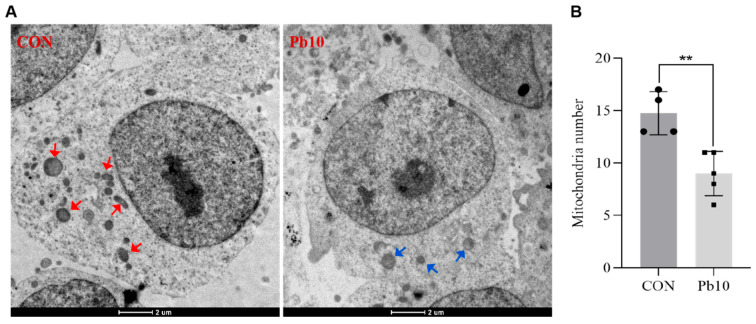
Representative TEM images of SH-SY5Y cells treated with 10 μM Pb for 24 h. (**A**) TEM images of control group and Pb10 group cells. Scale bar: 2 μm. (**B**) Statistical comparison of mitochondrial numbers between the two groups. Mitochondria exhibiting ultrastructural changes, such as a blurred structure or altered mitochondrial number (red arrows indicate mitochondria in control group cells; blue arrows indicate mitochondria in Pb10 group cells) (results are expressed as means ± SEM, *n* = 4. ** *p* < 0.01).

The CCK-8 assay results demonstrated a dose-dependent decrease in SH-SY5Y cell viability with increasing concentrations of lead acetate. Specifically, exposure to 10 μM lead acetate resulted in a statistically significant reduction in cell viability compared to the control group (*p* < 0.05, Figure 1A,B). Furthermore, transmission electron microscopy (TEM) images revealed substantial differences in mitochondrial ultrastructure between the control and the 10 μM lead-treated groups (Figure 2A,B). In the control group, mitochondria appeared normal with intact cristae, whereas the lead-treated group exhibited a significant decrease in mitochondrial number, accompanied by noticeable mitochondrial swelling and fragmentation (Figure 2A). Quantitative analysis of TEM images confirmed a significant reduction in the average number of mitochondria per cell in the lead-treated group compared to the control group (*p* < 0.01, Figure 2B). These findings collectively indicate that lead acetate adversely affects both the viability and mitochondrial integrity of SH-SY5Y cells, leading to mitochondrial morphological alterations and quantitative reduction. Based on these findings, 10 μM lead acetate was selected for all subsequent analyses, including whole transcriptome sequencing, to investigate the underlying molecular mechanisms of lead-induced cytotoxicity.

### 2.2. Effects of Lead Acetate on Mitochondrial Dynamics-Related Protein Expression

**Figure 3:**
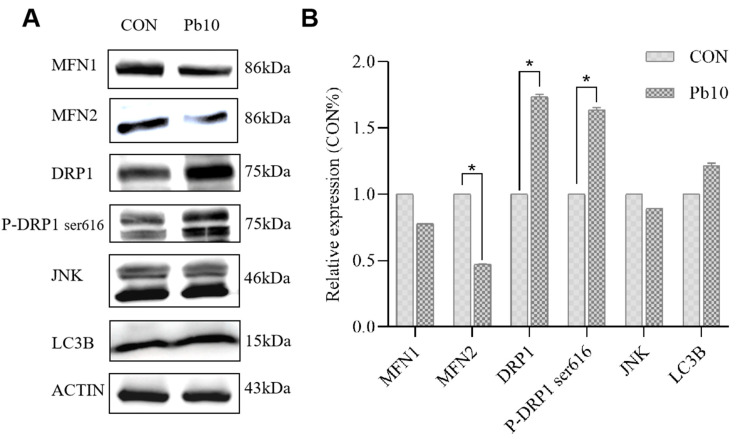
Lead acetate treatment effects on mitochondrial protein expression in SH-SY5Y cells. (**A**) Western blot analysis was performed to assess the expression levels of mitochondrial-related proteins. (**B**) Protein bands were visualized and quantified to determine the relative expression levels. (*n* = 3 for each group, * *p* < 0.05).

To further investigate the impact of lead acetate on mitochondrial dynamics, the protein expression levels of key mitochondrial fusion and fission proteins were analyzed by Western blot. Our results demonstrated that exposure to 10 μM lead acetate led to a significant decrease in the expression of MFN2, a protein primarily involved in mitochondrial outer membrane fusion (Figure 3A,B). Conversely, the total expression of DRP1, a key mediator of mitochondrial fission, and its phosphorylated form at serine 616 (P-DRP1 Ser616) were significantly increased in the lead-treated group compared to the control (Figure 3A,B). These findings clearly indicate an imbalance in mitochondrial dynamics, favoring fission over fusion, in response to lead acetate exposure. No statistically significant changes were observed in the protein expression of MFN1, total JNK, or LC3B (Figure 3A,B).

### 2.3. Differential Expression Analysis of ceRNAs

**Figure 4:**
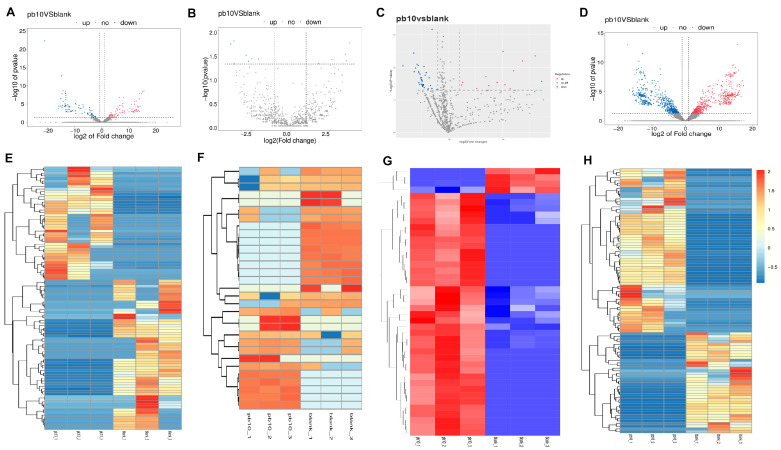
Differentially expressed RNAs. (**A**–**D**) Volcano plot of DE lncRNAs, DE circRNAs, DE miRNAs, and DE mRNAs. (**E**–**H**) Heatmap of DE lncRNAs, DE circRNAs, DE miRNAs, and DE mRNAs with the most obvious up-regulation and down-regulation, *p*-value < 0.05. In all panels, blue represents down-regulation and red represents up-regulation.

Differential expression analysis of whole transcriptome sequencing data revealed significant changes in the expression profiles of ceRNAs in lead-treated SH-SY5Y cells compared to the control group (Figure 4). Specifically, a total of 102 differentially expressed lncRNAs (DE lncRNAs) were identified, comprising 55 upregulated and 47 downregulated lncRNAs. We also found 31 differentially expressed circRNAs (DE circRNAs), with 13 upregulated and 18 downregulated. Additionally, 194 differentially expressed miRNAs (DE miRNAs) showed significant expression changes, including 21 upregulated and 173 downregulated miRNAs, suggesting a predominant downregulation of miRNAs. Furthermore, a total of 129 differentially expressed mRNAs (DE mRNAs) were identified, with 100 upregulated and 29 downregulated. These extensive changes in ceRNA expression collectively indicate a profound transcriptional reprogramming in SH-SY5Y cells in response to lead exposure, potentially impacting a wide range of cellular functions.

### 2.4. GO Analysis of Differentially Expressed ceRNAs

**Figure 5:**
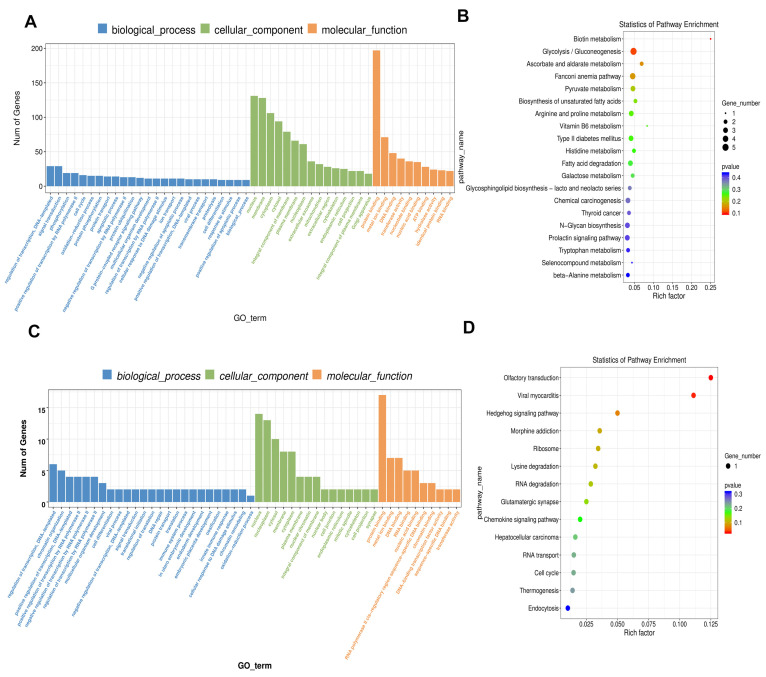

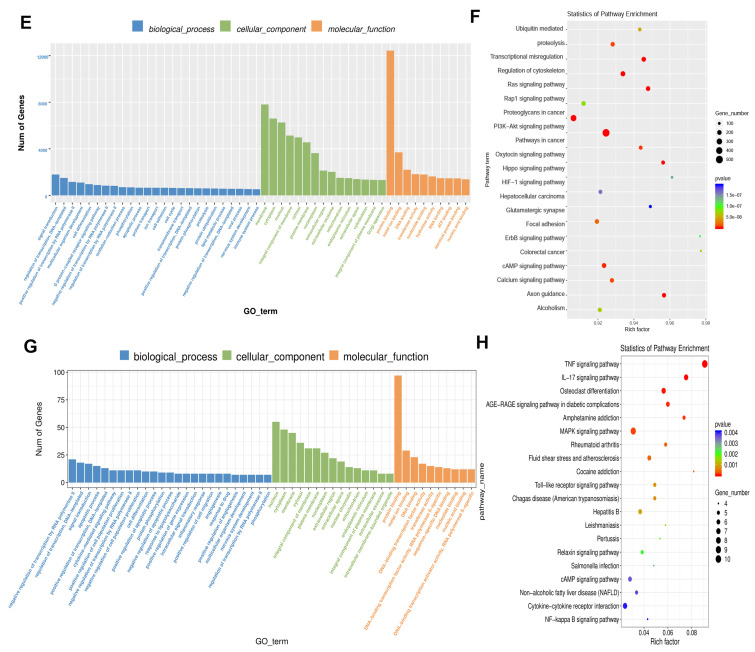
GO and KEGG enrichment analysis. (**A**,**B**) Enrichment annotation of DE lncRNAs. (**C**,**D**) Enrichment annotation of DE circRNAs. (**E**,**F**) Enrichment annotation of DE miRNAs. (**G**,**H**) Enrichment annotation of DE mRNAs.

The Gene Ontology (GO) enrichment analysis of differentially expressed ceRNAs uncovered several significant pathways associated with mitochondrial function. Notably, we identified enrichments in terms related to cellular response to oxidative stress, mitochondrial dynamics, negative regulation of apoptotic processes, and energy metabolism (Figure 5). These pathways underscore the critical roles of the identified RNA species in regulating mitochondrial integrity and function in response to lead. Notably, GO analysis consistently implicated pathways such as mitochondrial transport, mitochondrial membrane potential, and energy production across the differentially expressed lncRNAs, circRNAs, miRNAs, and mRNAs.

### 2.5. KEGG Enrichment Analysis

KEGG pathway analysis identified several critical signaling pathways implicated in lead-induced mitochondrial dysfunction, including the TNF signaling pathway, MAPK signaling pathway, cytokine–cytokine receptor interaction, NF-kappa B signaling pathway, Hippo signaling pathway, and Ferroptosis (Figure 5). Notably, these pathways were significantly enriched in the analysis of differentially expressed lncRNAs, circRNAs, miRNAs, and mRNAs, indicating their integral and collaborative role in regulating cellular metabolism, proliferation, and apoptosis in response to lead exposure.

### 2.6. PPI (Protein–Protein Interaction, PPI) Network Construction

**Figure 6:**
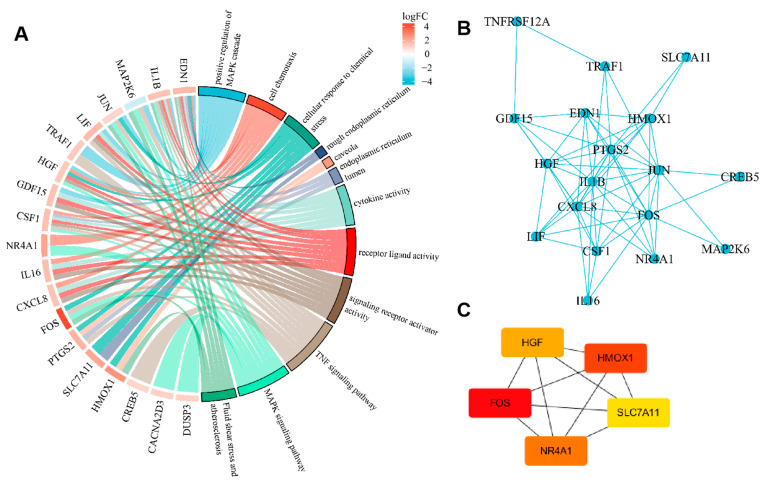
Protein–protein interaction network construction. (**A**) GO and KEGG enrichment analyses on the identified gene list. (**B**) PPI network between the identified proteins. (**C**) Identification of the five hub genes.

Based on the identified differentially expressed lncRNAs, circRNAs, miRNAs, and mRNAs, we predicted the potential ceRNA interactions. Specifically, miRNA–mRNA interactions were predicted using TargetScan and miRBase databases, while lncRNA/circRNA–miRNA interactions were predicted using established bioinformatics tools (e.g., miRcode, LncBase, CircBase, etc.). Subsequently, we constructed a comprehensive ceRNA network to identify core genes potentially involved in lead-induced neurotoxicity. This network analysis, followed by GO and KEGG enrichment analyses on the hub genes identified from the network, revealed significant biological processes and pathways pertinent to our investigation (Figure 6A). To further explore protein-level interactions, we also constructed a PPI network from the differentially expressed mRNAs (Figure 6B). This integrated bioinformatics approach led to the identification of five key core genes: *SLC7A11*, *FOS*, *HMOX1*, *HGF*, and *NR4A1* (Figure 6C). These core genes are implicated in crucial cellular processes such as ferroptosis (*SLC7A11*), oxidative stress response (*HMOX1*), transcriptional regulation (*FOS*, *NR4A1*), and cell growth/survival (*HGF*), aligning with the observed lead-induced cellular damage and mitochondrial dysfunction.

### 2.7. DE lncRNA/DE circRNA–DE miRNA–DE mRNA Network Construction

**Figure 7:**
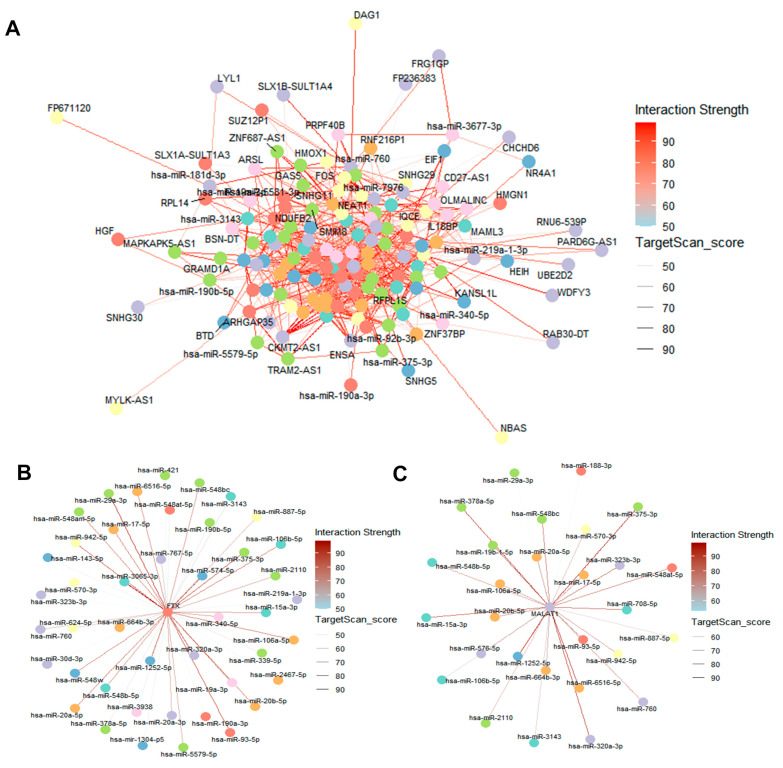
ceRNA network construction. (**A**) DE lncRNA–DE miRNA–DE mRNA network. (**B**) *lncFTX*–DE miRNAs network. (**C**) *lncMALAT1*–DE miRNAs network. The node color represents the degree of correlation. The line color represents the TargetScan score.

**Figure 8:**
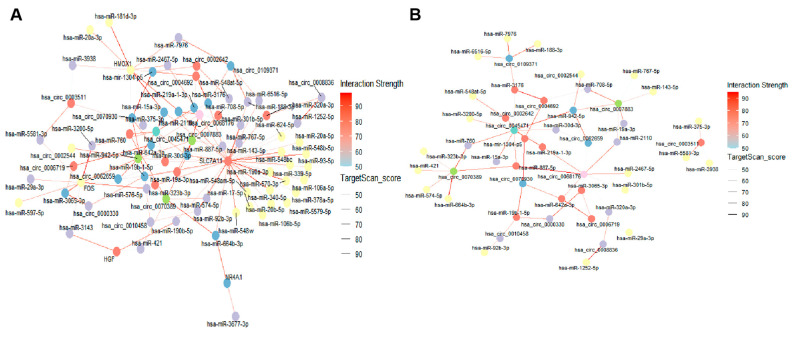
DE circRNA–DE miRNA–DE mRNA network construction. (**A**) DE circRNAs–DE miRNAs–DE mRNAs network. (**B**) Several core circRNAs–DE miRNAs network. The node color represents the degree of correlation. The line color represents the TargetScan score.

Building upon the identified core genes and differentially expressed non-coding RNAs, we constructed a comprehensive ceRNA network involving the five key core genes (*SLC7A11*, *FOS*, *HMOX1*, *HGF*, and *NR4A1*) along with DE lncRNAs, DE circRNAs, and DE miRNAs to elucidate their intricate regulatory interactions (Figure 7A and Figure 8A). This network demonstrates how lncRNAs and circRNAs act as miRNA sponges to regulate the expression of target mRNAs, including our identified core genes. Figure 8B provides illustrative subgraphs that clearly delineate the molecular relationships among these components, highlighting key regulatory axes. Specifically, Figure 7B,C illustrate representative lncRNA–miRNA subnetworks for two lncRNAs, *FTX* and *MALAT1*, respectively, highlighting their interactions with corresponding miRNAs. For instance, *FTX* and *MALAT1* are well-known lncRNAs implicated in various cellular processes and diseases, including neuronal function and stress responses [33,34,35,36], suggesting their potential involvement in lead-induced neurotoxicity through miRNA sponging. The entire ceRNA network was visualized using R (version 4.4.2), effectively illustrating the complex regulatory relationships among these RNA molecules and the core genes in response to lead exposure.

### 2.8. Validation of Key Genes

**Figure 9:**
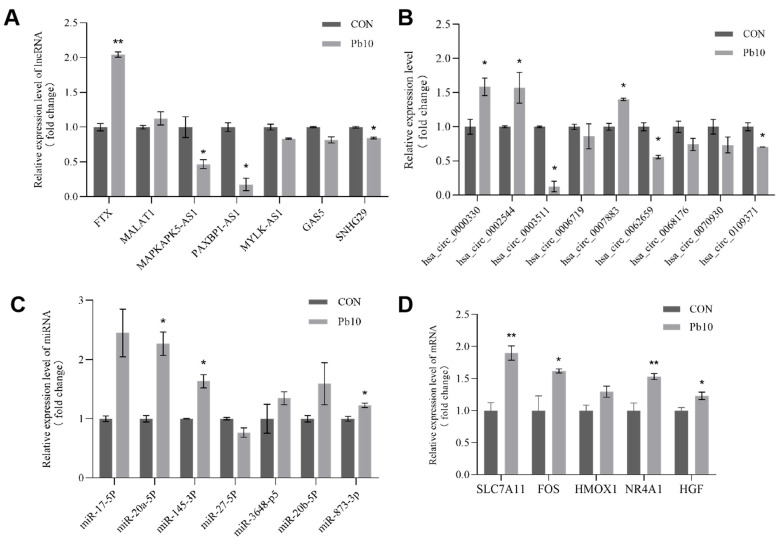
qRT-PCR validation of DE ceRNAs in CON and 10μM Pb group. (**A**) DE lncRNAs. (**B**) DE circRNAs. (**C**) DE miRNAs. (**D**) DE mRNAs. (*n* = 3, * *p* < 0.05, ** *p* < 0.01).

To validate the accuracy of the RNA-Seq data and confirm the expression changes in key regulatory molecules, qRT-PCR was conducted for selected lncRNAs, circRNAs, miRNAs, and core mRNAs identified from the ceRNA network. Among the validated lncRNAs, *FTX* was found to be significantly upregulated, while *MAPKAPK5-AS1*, *PAXBP1-AS1*, and *SNHG29* were significantly downregulated (Figure 9A). For circRNAs, *hsa_circ_0000330*, *hsa_circ_0002544*, and *hsa_circ_0007883* showed significant upregulation, whereas *hsa_circ_0003511*, *hsa_circ_0062659*, and *hsa_circ_0109371* exhibited significant downregulation (Figure 9B). Additionally, the validated miRNAs included *miR-20a-5p*, *miR-145-3p*, and *miR-873-3p*, all of which demonstrated significant upregulation (Figure 9C). Crucially, the expression levels of all five core mRNAs were also validated by qRT-PCR, *SLC7A11*, *FOS*, *HGF*, and *NR4A1* were found to be significantly upregulated in the lead-treated group compared to the control group (Figure 9D). The expression patterns of all validated lncRNAs, circRNAs, miRNAs, and mRNAs were consistent with the RNA-Seq results, thereby confirming the reliability and robustness of our transcriptomic analysis and the predicted ceRNA network.

## 3. Discussion

This study elucidates that lead (Pb) exposure significantly impacts mitochondrial quantity and morphology in SH-SY5Y cells, and, crucially, reveals potential regulatory mechanisms involving ceRNA networks. Our transmission electron microscopy (TEM) results provided compelling visual evidence of these detrimental effects, revealing a marked reduction in mitochondrial quantity, alongside pronounced mitochondrial swelling and cristae disruption in SH-SY5Y cells treated with 10 μM lead acetate for 24 h. These observations are notably consistent with previous studies reporting similar structural alterations under lead toxicity, underscoring the universal nature of these morphological changes [37]. The observed mitochondrial damage aligns with established mechanisms of lead neurotoxicity, which include direct membrane damage, increased permeability, calcium dysregulation, and subsequent release of pro-apoptotic factors, ultimately triggering apoptosis [38].

These ultrastructural changes are characteristic indicators of mitochondrial dysfunction and are often associated with altered mitochondrial dynamics [8]. Our subsequent Western blot analysis provided crucial mechanistic insights into these observations, demonstrating a significant shift in the balance of mitochondrial fusion and fission proteins. We found a notable decrease in the expression of the major mitochondrial fusion protein MFN2 while simultaneously observing a significant increase in the expression of the mitochondrial fission protein DRP1 and its activated phosphorylated form (P-DRP1 at Ser616). This observed imbalance, favoring excessive fission over fusion, is a well-established driver of mitochondrial fragmentation, loss, and ultimately, cellular dysfunction [39,40]. These findings indicate that lead-induced disturbance of mitochondrial fission-fusion balance is a key contributor to mitochondrial dysfunction in neurotoxicity. However, the molecular mechanisms, particularly the roles of non-coding RNAs in this process, require further exploration.

Recent studies have increasingly highlighted the pivotal roles of various lncRNAs in regulating mitochondrial function, strongly suggesting their potential involvement in the complex mechanisms of lead (Pb) toxicity. In line with this, our transcriptome analysis identified several differentially expressed lncRNAs, and we further validated key candidates, including *FTX*, *MAPKAPK5-AS1*, *PAXBP1-AS1*, and *SNHG29*, whose expression changes were consistent with our RNA-Seq data. Notably, lncRNA *FTX* inhibits ferroptosis in magnesium-free-induced hippocampal neurons via the *miR-142-5p/GABPB1* axis [33] and protects against cerebral ischemia–reperfusion injury by enhancing neuronal proliferation and reducing apoptosis through the *miR-186-5p/MDM4* pathway [41]. Similarly, *MAPKAPK5-AS1* has been shown to enhance mitochondrial function and protect against oxidative damage by sequestering *miR-146a-3p* to regulate *SIRT1* [42]. Although the roles of *PAXBP1-AS1* and *SNHG29* under lead exposure are less well-studied, their differential expression and known involvement in cell proliferation and apoptosis suggest they may contribute to lead-induced neurotoxicity [43]. These findings indicate that the identified lncRNAs may participate in regulating mitochondrial function and cell survival in response to lead exposure, pointing to potential therapeutic targets for mitigating lead-induced neuronal damage.

Our study also identifies several circRNAs with differential expressions in Pb-treated SH-SY5Y cells, and we validated the expression patterns of *hsa_circ_0000330*, *hsa_circ_0002544*, *hsa_circ_0007883*, *hsa_circ_0003511*, *hsa_circ_0062659*, and *hsa_circ_0109371*. While some of these circRNAs, such as *hsa_circ_0007883*, have been previously linked to cancer progression (e.g., ZEB1 regulation in ovarian cancer), their specific roles in lead-induced neurotoxicity and mitochondrial function require further elucidation [44]. Although some of these circRNAs have been previously associated with disease processes, their specific roles in lead-induced neurotoxicity and mitochondrial function remain unclear. Given the critical role of circRNAs as miRNA sponges and their potential in regulating gene expression, our findings suggest that these differentially expressed circRNAs may contribute to lead-induced mitochondrial damage by modulating the ceRNA network.

Our comprehensive ceRNA network analysis, integrated with PPI network construction, led to the identification of five key core mRNAs: *SLC7A11*, *FOS*, *HMOX1*, *HGF*, and *NR4A1*. Crucially, our qRT-PCR validation confirmed that all five of these core genes were significantly upregulated in lead-treated SH-SY5Y cells, aligning with their potential roles in cellular response to lead-induced stress. These genes are integral to the enriched pathways we identified, such as ferroptosis and oxidative stress. For example, *SLC7A11* is known to participate in ferroptosis and oxidative stress regulation, and its upregulation may be mediated by specific miRNAs within the ceRNA network [45]. *HMOX1* is associated with cellular adaptation to oxidative stress, while *FOS*, *HGF*, and *NR4A1* are involved in regulating stress responses, cell survival, and neuroprotection [46,47]. The consistent upregulation of these core genes highlights their central roles in the cellular response to lead-induced neurotoxicity, potentially through ceRNA network regulation.

In summary, our study uncovers the central role of ceRNA networks in lead-induced neurotoxicity, providing a comprehensive transcriptomic landscape of SH-SY5Y cells under lead exposure. We demonstrate that mitochondrial dysfunction and neurotoxicity are closely linked to significant changes in non-coding RNA and core gene expression (*SLC7A11*, *FOS*, *HMOX1*, *HGF*, *NR4A1*), highlighting these genes as critical mediators of cellular stress responses. Although limited by the use of in vitro models, our findings establish a novel molecular framework for understanding the regulatory mechanisms underlying lead neurotoxicity and point to ceRNA axes as promising targets for future diagnostic and therapeutic strategies. Further validation in functional and in vivo studies will be essential to fully elucidate their clinical potential.

## 4. Methods

### 4.1. Cell Culture and Lead Treatment

Human neuroblastoma SH-SY5Y cells were maintained in Dulbecco’s Modified Eagle Medium (DMEM; Gibco, Grand Island, NY, USA) supplemented with 10% heat-inactivated fetal bovine serum (FBS; Gibco) and 1% penicillin-streptomycin (Gibco). Cells were cultured at 37 °C in a humidified atmosphere containing 5% CO_2_. For experiments, SH-SY5Y cells were seeded into 6-well plates at a density of 1 × 10^6^ cells per well and allowed to adhere overnight. Subsequently, cells were exposed to 10 μM lead acetate [Pb(CH_3_COO)_2_] for 24 h. The exposure concentration (10 μM) and duration (24 h) were selected based on our preliminary experiments and published literature, which demonstrated that these conditions induce significant cellular and molecular responses without causing excessive cell death. Untreated cells cultured under identical conditions served as the control group. All experiments were performed in triplicate to ensure reproducibility.

### 4.2. Cell Counting Kit-8

To assess the effects of lead acetate on the viability of SH-SY5Y cells, a Cell Counting Kit-8 (CCK-8; Beyotime, Shanghai, China) assay was performed according to the manufacturer’s instructions. Cells were seeded into 96-well plates at a density of 1 × 10^4^ cells per well and allowed to adhere for 24 h at 37 °C in a humidified atmosphere with 5% CO_2_. After adherence, cells were exposed to various concentrations of lead acetate (0, 1, 5, 10, 20, and 50 μM) for an additional 24 h. Following treatment, 10 μL of CCK-8 solution was added to each well, and the plates were subsequently incubated for 1 h at 37 °C. The absorbance at 450 nm was measured using a microplate reader (Tecan, Männedorf, Switzerland). Cell viability was expressed as a percentage relative to the untreated control group. All experiments were performed in triplicate to ensure reproducibility.

### 4.3. Transmission Electron Microscopy (TEM)

SH-SY5Y cells were cultured under standard conditions and treated with 10 μM lead acetate for 24 h, while control cells were maintained without lead exposure. Following treatment, cells were harvested and fixed with 2.5% glutaraldehyde in 0.1 M phosphate buffer (pH 7.4) for 2 h at 4 °C, followed by post-fixation with 1% osmium tetroxide (Ted Pella, Redding, CA, USA) for 1 h at 4 °C. After thorough washing, cells were dehydrated through a graded ethanol series and embedded in epoxy resin (SPI, West Chester, PA, USA) according to standard protocols. Ultrathin sections (~70 nm) were cut using an ultramicrotome (Leica, Wetzlar, Germany) and subsequently stained with uranyl acetate and lead citrate. Images were acquired using a transmission electron microscope (TEM; JEOL JEM-1400, Tokyo, Japan). Mitochondrial numbers were quantified from randomly selected fields using ImageJ software (V1.54e, NIH, Bethesda, MD, USA). Statistical analysis was performed using GraphPad Prism Software (V8.0.2, San Diego, CA, USA). All experiments were carried out in triplicate.

### 4.4. Western Blotting Assay

Cells were washed twice with ice-cold phosphate-buffered saline (PBS) and lysed in cell lysis buffer (10 mM Tris-HCl, pH 7.4, 1% SDS, 1 mM Na_3_VO_4_) supplemented with protease and phosphatase inhibitors (Beyotime). The lysates were sonicated on ice and then denatured by heating at 100 °C for 5 min. Protein concentrations were determined using a BCA Protein Assay Kit (Beyotime) according to the manufacturer’s instructions. Equal amounts of protein were separated by SDS-polyacrylamide gel electrophoresis (SDS-PAGE) and transferred onto PVDF membranes (Bio-Rad, Hercules, CA, USA).

Membranes were blocked with 5% non-fat milk in TBST for 1 h at room temperature and then incubated overnight at 4 °C with primary antibodies against MFN1, MFN2, DRP1, p-DRP1 (Ser616), JNK, LC3B, and β-Actin as an internal control (all from Cell Signaling Technology, Beverly, MA, USA). After washing, membranes were incubated with alkaline phosphatase-conjugated secondary antibodies for 1 h at room temperature. Protein bands were visualized using the ECF Western Blotting System (Amersham, Piscataway, NJ, USA). Densitometric analysis was performed using ImageJ V1.54e. All experiments were conducted in triplicate.

### 4.5. RNA Extraction and Sequencing

Total RNA was extracted using the TRIzol reagent (Invitrogen, Carlsbad, CA, USA) following the manufacturer’s instructions. RNA quality and integrity were assessed using an Agilent 2100 Bioanalyzer (Agilent Technologies, Santa Clara, CA, USA), and samples with RNA integrity number (RIN) > 7 were used for sequencing. RNA-Seq libraries were prepared using the NEBNext Ultra RNA Library Prep Kit for Illumina (New England Biolabs, Ipswich, MA, USA) according to the manufacturer’s protocol. Sequencing was performed on an Illumina HiSeq 2500 platform, generating 150 bp paired-end reads.

### 4.6. Data Analysis

Raw sequencing reads were subjected to quality control using FastQC (v0.11.8) and trimmed for adapters and low-quality bases using Trimmomatic (v0.39). Clean reads were aligned to the human genome (GRCh38) using HISAT2 (v2.1.0). Read counts for each gene were obtained using feature Counts (v1.6.3), and differential expression analysis was performed using DESeq2 (v1.22.2) in R. Differentially expressed RNAs were identified with |log2FoldChange| > 1 and adjusted *p*-value < 0.05. Heatmap analysis was performed on the differentially expressed ceRNAs to visualize expression patterns between the control and lead treatment groups.

### 4.7. Functional Enrichment Analysis

Functional enrichment analysis was performed using DAVID (v6.8) and KEGG pathway analysis to identify significantly enriched biological processes and pathways associated with the differentially expressed RNAs. Pathways with an adjusted *p* < 0.05 were considered significant.

### 4.8. PPI and ceRNA Network Construction

TargetScan (https://www.targetscan.org/vert_80/, accessed on 10 November 2024) and miRBase (https://www.mirbase.org/, accessed on 15 November 2024) were used to predict the interaction relationships among lncRNAs, circRNAs, miRNAs, and mRNAs. The PPI and ceRNA network was constructed and visualized using Cytoscape software (V3.10.3, Cytoscape Consortium, San Diego, CA, USA). The top five hub mRNAs were identified through Cytoscape software’s cytoHubba plugin (V0.1, National Taiwan University, Taipei, Taiwan), allowing for the construction of a visualized ceRNA network demonstrating the targeted relationships among these molecules.

### 4.9. Quantitative Real-Time PCR

Selected key genes from the ceRNA network were validated by qRT-PCR. Total RNA was reverse-transcribed using the PrimeScript RT reagent Kit (Takara, Kyoto, Japan), and qRT-PCR was performed using SYBR Premix Ex Taq II (Takara) on a Fast Real-Time PCR instrument (ABI-7500, Thermo Fisher Scientific, Waltham, MA, USA). Relative gene expression was calculated using the 2^−ΔΔCt^ method, with GAPDH as the internal control.

### 4.10. Statistical Analysis

Statistical analyses and data visualization were conducted using GraphPad Prism 8.0.2. Descriptive statistics, including means and standard deviations, were calculated for all experimental groups. To compare differences between two groups, Student’s *t*-test was utilized, while one-way ANOVA followed by Tukey’s post hoc test was applied for comparisons involving more than two groups. Additionally, correlation analyses were performed using Pearson’s correlation coefficient to explore relationships between variables. Graphical representations of the data were created using Prism’s built-in graphing capabilities, ensuring clarity and accuracy in the presentation of results. All data were presented as mean ± standard error of the mean (SEM). The significance level was set at *p* < 0.05.

## 5. Conclusions

Our study reveals that lead exposure significantly reduced mitochondrial quantity and impaired their morphology in SH-SY5Y cells. Transcriptomic analysis identified differentially expressed lncRNAs, circRNAs, miRNAs, and mRNAs, highlighting pathways associated with mitochondrial function. A ceRNA network was constructed, focusing on key hub genes (*SLC7A11*, *FOS*, *HMOX1*, *HGF*, *NR4A1*) integral to cellular oxidative stress response and mitochondrial health. These findings highlight the central role of ceRNA-mediated regulatory mechanisms in lead-induced mitochondrial dysfunction and neurotoxicity. Our work provides novel insights into the molecular pathways underlying lead neurotoxicity and establishes a basis for the development of targeted therapeutic strategies to mitigate lead-induced neuronal damage.

## Data Availability

The raw data supporting the conclusions of this article will be made available by the authors on request.

## Abbreviations

ATP	Adenosine triphosphate
GTPases	Guanosine triphosphatases
DRP1	Dynamin-related protein 1
JNK	c-Jun N-terminal kinase
LC3B	Microtubule-associated protein 1 light chain 3 beta
ceRNA	Competing endogenous RNA
SH-SY5Y	Human neuroblastoma cell line
DE lncRNAs	Differentially expressed lncRNAs
ncRNAs	Non-coding RNAs
TEM	Transmission electron microscopy
qRT-PCR	Quantitative real-time PCR
PPI	Protein–Protein Interaction
